# *Trichuris muris* Model: Role in Understanding Intestinal Immune Response, Inflammation and Host Defense

**DOI:** 10.3390/pathogens10080925

**Published:** 2021-07-22

**Authors:** Yeganeh Yousefi, Sabah Haq, Suhrid Banskota, Yun Han Kwon, Waliul I. Khan

**Affiliations:** 1 Farncombe Family Digestive Health Research Institute, McMaster University Health Sciences Centre Room 3N7, 1280 Main St. W, Hamilton, ON L8N 3Z5, Canada; yeganeh.yousefi.90@gmail.com (Y.Y.); haqs4@mcmaster.ca (S.H.); banskots@mcmaster.ca (S.B.); yyoon90@gmail.com (Y.H.K.); 2 Department of Pathology and Molecular Medicine, McMaster University, 1200 Main St. W, Hamilton, ON L8N 3Z5, Canada

**Keywords:** intestinal helminth, *Trichuris muris*, immune response, host–parasite interaction, host defense, epithelial cells, goblet cells, enteroendocrine cells, smooth muscle cells

## Abstract

Several parasites have evolved to survive in the human intestinal tract and over 1 billion people around the world, specifically in developing countries, are infected with enteric helminths. *Trichuris trichiura* is one of the world’s most common intestinal parasites that causes human parasitic infections. *Trichuris muris*, as an immunologically well-defined mouse model of *T. trichiura*, is extensively used to study different aspects of the innate and adaptive components of the immune system. Studies on *T. muris* model offer insights into understanding host immunity, since this parasite generates two distinct immune responses in resistant and susceptible strains of mouse. Apart from the immune cells, *T. muris* infection also influences various components of the intestinal tract, especially the gut microbiota, mucus layer, epithelial cells and smooth muscle cells. Here, we reviewed the different immune responses generated by innate and adaptive immune components during acute and chronic *T. muris* infections. Furthermore, we discussed the importance of studying *T. muris* model in understanding host–parasite interaction in the context of alteration in the host’s microbiota, intestinal barrier, inflammation, and host defense, and in parasite infection-mediated modulation of other immune and inflammatory diseases.

## 1. Introduction

Intestinal parasites are one of the most important parasites in terms of their widespread prevalence, and they have major socioeconomic impacts on both developing and developed countries by affecting human and animal well-being, productivity, and agriculture. Among the intestinal parasites, intestinal helminth infections are the most prevalent parasites, and they occur through contact with parasite eggs and larvae. It is estimated that about 2 billion people worldwide are infected with helminths [1]. *Trichuris trichiura* is a soil-transmitted helminth, and recent estimates suggest that there are approximately 465 million people worldwide with *T. trichiura* infection [2,3,4]. Infection with *T. trichiura* is associated with adverse health consequences in humans, with the majority being children [5,6].

*T. muris,* a murine pathogen, shares extensive homology at genomic, transcriptomic and morphological levels to *T. trichiura* and is extensively used as a laboratory mouse model for *T.* *trichiura* [7]. *T. muris*, with its unique advantage of producing heterogeneous immunological outcomes in different mouse strains, is a widely used model for understanding host–parasite interactions. Models of helminth infection are of immense importance in exploring the pathology and pathophysiology of many gastrointestinal disorders [8]. Due to well-defined immunity and biology, the *T. muris* model is widely used for understanding inflammatory changes, epithelial barrier function, immune responses and host defense mechanisms in intestinal infection and inflammation. Recent evidence indicates that *T. muris* shares its environment with gut microbiota and develops bidirectional interactions with the microbes. These interactions are also immunologically important since they involve the host immune system and therefore have an impact on host defense mechanisms. This review focuses on the importance of the *T. muris* model in understanding intestinal immune response, inflammation and host defense with a view to illuminating the utility of this model in studying the pathophysiology of various immune and inflammatory disorders.

## 2. *T. muris* Model

### 2.1. Life Cycle of T. muris

The life cycle of *T. muris* starts with the ingestion of infective eggs released in feces of infected hosts. Upon ingestion, eggs migrate toward the intestine and accumulate in the cecum. About 90 min after egg ingestion, the eggs hatch, followed by L1 larval development in the cecum and the colon of infected mice. The L1 larvae then molt three times into L2 (9–11 days post-infection (p.i.)), L3 (17 days p.i.) and L4 (22 days p.i.). Larval molting activity durations vary in different host strains. After larval development (by day 32 p.i.), parasites move toward the gut lumen, and the adult form of *T. muris* is detected in the cecum and proximal colon [9,10,11].

The intracellular attachment of the anterior head of *T. muris* to the cytoplasm of epithelial cells forms “syncytial tunnels”. Since lateral cell walls are not stabilized by cytoskeleton and cell junctions, *T. muris* entry into the epithelial cells causes rupture of lateral walls. In contrast, stabilizing actin cytoskeleton and cell junctions prevent rupture in apical and basal surfaces of the epithelial cells following worm entry [9,10,12]. It was reported that bacillary cells, the specialized cellular structure of some helminths including *T. muris*, facilitate the establishment of an intracellular niche for *T. muris* through glucose absorption [13]. Eventually, mature adult worms produce 1000–2000 unembryonated eggs per day, which are shed into host feces. After approximately 2 months, released eggs change into embryonated and infective eggs and then are able to infect new hosts [9,11].

Successful completion of the life cycle of *T. muris* is heavily dependent on several factors, including genetic background, strains and gender of the mouse, and the infective dose and the strain of *T. muris*. Depending on the mouse strain, there are two different immune response phenotypes, resistant and susceptible, against *T. muris* infection [14]. More than 70% of mouse strains such as BALB/k, BALB/c, C57BL/6 (high dose of infection), are resistant to *T. muris* infection and eliminate the worms approximately by day 32 p.i. [10,15] via a Th2 dependent response [10]. In contrast, the remaining mouse strains such as AKR, B10Br, and C57BL/6 (low dose of infection) develop a Th1 immune response and become susceptible to chronic *T. muris* infection [10,15].

It was shown that differences in the H-2 alleles of the major histocompatibility complex (MHC) in mice with a similar genetic background, B10, influence CD4^+^ T cell activation and thus ultimately result in a distinct phenotype in response to *T. muris* infection [14,16]. Furthermore, some aspects of the life cycle of *T. muris* might differ based on mice gender due to the different immunomodulatory effects of host-derived sex steroid hormones [17].

The infective dose of *T. muris* eggs can impact the life cycle and immune response outcomes by changing CD4^+^ T cell polarization [18]. The lower doses of antigen stimulate Th1 immune responses seen in susceptible mice, while a high infection dose is linked to Th2 immune responses [19]. As an example, a low dose of infection (<40 eggs) in resistant mice such as BALB/k shifts immune responses toward Th1 immunity, rendering mice susceptible [18]. Additionally, various strains of *T. muris* namely S (Sobreda), E (Edinburgh), and J (Japan) strains have differences in their immune responses. B10.BR, CBA, C57BL/6 and C57BL/10 mice are resistant to infection with E and J strains but are susceptible to S strain [20,21].

### 2.2. Immune Responses against T. muris Infection

**Figure 1:**
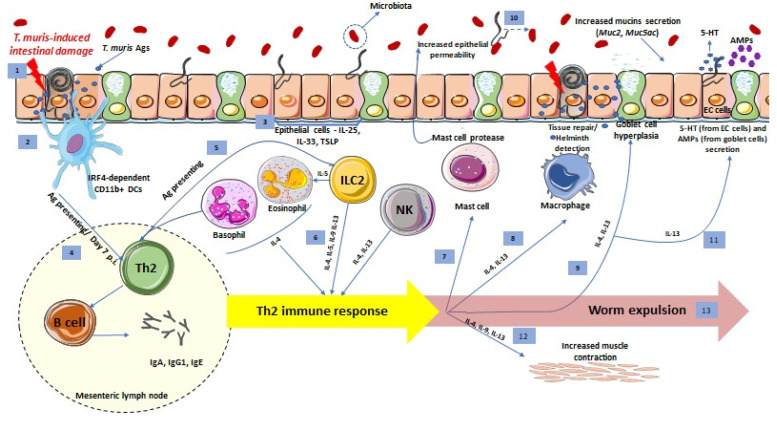
Host intestinal immune response against *T. muris* infection. Representative schematic of the helminth detection and immune response induction. (1) *T. muris* larvae breach the intestinal epithelium and release Ags. (2) IRF4-dependent CD11b^+^ DCs up-take *T. muris* Ags (3) Upon *T. muris* invasion, intestinal epithelial cells produce alarmins that recruit immune cells. (4) DCs present *T. muris* Ags to adaptive immune cells in the mesenteric lymph node on day 7 p.i. and activate Th2 cells (the main source of Th2 cytokines). Th2 cells stimulate B cells that synthesize and secret IgA, IgG1 and IgE. (5) Innate immune cells also present Ags to adaptive immune cells in the mesenteric lymph node. (6) Innate immune cells (basophils, eosinophils, ILC2s and NK cells) release Th2 cytokines and promote Th2 immune response. The effects of Th2 immune response are: (7) Activation of mast cells to release mast cell protease, which increases epithelial cell permeability, (8) development of alternatively activated macrophages, which cause tissue repair and helminth detection, (9) goblet cell hyperplasia and increased mucins (*Muc2* and *Muc5ac*) secretion. (10) *T. muris* affect microbiota composition, which subsequently can influence mucin secretion and barrier function, (11) increase production of 5-HT and AMPs from EC cells and goblet cells, respectively, and (12) increase smooth muscle contraction. (13) All these Th2 responses lead to *T. muris* expulsion.

It has been shown that both innate and adaptive immunity components are involved in generating protective responses against *T. muris* infection (Figure 1). However, some immunological mechanisms generated in response to *T. muris* infection are still poorly understood [9]. Studies on intestinal helminth infections are in agreement that innate immune cells and cytokines are of crucial importance for the induction of the adaptive Th2 immune response [22].

#### 2.2.1. Innate Immune Response

##### Macrophages

Macrophages, the most abundant mononuclear phagocytes found in tissues, are recognized as essential players in generating an immune response against *T. muris* infection. Upon parasite invasion, alternatively activated macrophages (AAMs) are induced and play a role in amplifying the initial Th2 immune cascade promoted by the epithelial cells and type 2 innate lymphoid cells (ILC2s) (Figure 1) [23]. Reportedly, AAMs are likely to contribute to tissue repair since their population increases after worm expulsion [24,25]. AAMs express mannose receptors (MRs), which are important pattern recognition receptors (PRRs) involved in the detection of helminths and in initiating immune responses [23]. The in vitro stimulation of bone-marrow-derived macrophages (BMDMs) with *T. muris* excretory/secretory products (ESPs) led to the production of multiple cytokines such as IL-6 and IL-10 by interacting with MRs. However, experiments on mannose-deficient mice demonstrated that the absence of MRs on AAMs had no impact on parasite expulsion. These findings imply that MRs are not essential for the generation of an immune response leading to the expulsion of *T. muris*, and other alternative PRRs expressed on macrophages might be contributing in developing protective immune responses against *T. muris* antigens (Ags) [26].

##### Dendritic Cells (DCs)

DCs act as bridges between innate and adaptive immunity and play a role in the development of Th2 immunity [22]. In mice with resistant and susceptible immune responses to *T. muris* infection, different kinetics of DC responses have been observed. In the early days of infection, resistant mice have a higher number of DCs in the sites of infection compared to susceptible mice, and these recruited DCs are mature by day 7 p.i. in resistant mice. In contrast, a delayed DC response has been discovered in susceptible mice, and the number of DCs in these mice does not increase by day 13 p.i. Moreover, the maturation status of DCs is different between resistant and susceptible mice during *T. muris* infection. DCs in resistant mice have elevated levels of CD80/86, MHC class II and CCR7 expression, and reduced endocytic activity compared to DCs in susceptible mice, suggesting that resistant mice’s DCs mature quicker [27].

In resistant mice with *T. muris* infection, colonic DCs form transepithelial dendrites, which are not typically seen in naïve mice. These colonic transepithelial dendrites provide a direct sample of the luminal Ags to naïve T cells. Dendrites also can assist the transfer of Ags through colonic epithelial cells by pinocytosis (Figure 1). Therefore, in resistant mice, rapid DC mobilization to the epithelium can boost interactions between epithelial cells and DCs, enabling faster and more effective uptake of Ags and thus leading to a more efficient immune response. The presence of transepithelial dendrites only in mice with an efficient immune response against *T. muris* infection suggests that these dendrites are critical in mediating the development of resistance to infection [27].

On day 7 p.i. antigen-bearing DCs migrate to mesenteric lymph nodes (MLNs) where naïve T cells are located to stimulate T cell polarization, leading to rapid parasite removal [27]. There are three subsets of DCs based on CD103 and CD11b expression: CD103^+^CD11b^−^, CD103^+^CD11b^+^, and CD103^−^CD11b^+^. Among subsets of colonic DCs, CD11b^+^ DCs have a key part in enhancing Th2 responses during *T. muris* infection [28]. The expansion and survival of DC subsets are dependent on either interferon regulatory factor 4 or 8 (IRF4 or IRF8) [29]. IRF4-dependent CD11b^+^ DCs have a role in the activation of Th2 immunity and therefore in the development of resistant-associated immune responses during *T. muris* infection. Conversely, IRF8-dependent CD103^+^ DCs are related to *T. muris* chronicity as they facilitate Th1 immunity [28,30].

In addition, it has been proposed that epithelial-derived factors drive the responses of DCs, since the initial response of DCs in resistant mice is linked to increases in epithelial cell-derived chemokines such as CCL5 and CCL20 [27].

The initial events regulating immune responses to *T. muris* are poorly known. Around the time the worm is expelled (day 14 after infection), CD4+ T helper cells accumulate in the colonic epithelium. In resistant mice, however, the initial recruitment of DCs occurs several days before the production of adaptive immune responses [27,31,32]. These findings suggest that DCs may have an important role in determining and generating effective immune responses against *T. muris* infection, in particular during the initial stages of infection.

##### Basophils

Basophils are other innate immune cells that strengthen defensive immune responses during infection with *T. muris*. Epithelium-derived thymic stromal lymphopoietin (TSLP) induces basophil expansion in response to *T. muris* infection (Figure 1). It was also demonstrated that basophils expressing MHC class II are able to initiate Th2 responses upon *T. muris* infection and these cells are a major initial source for IL-4 in *T. muris* infection [9,33,34].

Perrigoue et al. demonstrated that CD4^+^ T cells in basophil-deficient mice fail to differentiate into Th2 subsets during *T. muris* infection. Thus, loss of basophils leads to reduced levels of IL-4, decreased goblet cell numbers, and impaired worm clearance in *T. muris-*infected mice [35]. Furthermore, it was recently shown that Notch signaling enhances basophil-associated immune responses during *T. muris* infection, and mice with basophil-specific inhibition of Notch signaling have decreased gene expression in basophils and therefore impaired worm expulsion [36].

In contrast, the development of Th2 responses was not inhibited in basophil-deficient mice upon infection with other helminths such as *Nippostrongylus brasiliensis* and *Schistosoma mansoni* [37,38,39]. These contrasting findings in different parasitic infections illustrate that the response of host basophils differs tremendously among distinctive helminthic infections. Thus, the role of basophils in promoting Th2 immune responses is not yet clear and needs further investigation.

##### Eosinophils

Increased number of eosinophils in the colon and mesenteric lymph nodes (MLNs) has been documented in helminth infections, including *T. muris* [40]. The recruitment of eosinophils in colonic mucosa during *T. muris* infection is regulated by IL-5 and the chemokine CCL11 [40] (Figure 1). *T. muris-*infected CCL11 KO mice have decreased colon eosinophil numbers, and there is a complete absence of eosinophils in the double CXCL11 and IL-5 KO *T. muris-*infected mice [11]. It was shown that in *T. muris-*infected resistant BALB/c mice, there was accumulation of eosinophils in MLNs. Accumulation of eosinophils in MLNs was initiated during the second week of infection and peaked during parasite expulsion. In contrast, there was a comparably late and modest increase in eosinophil numbers in the MLNs of infected susceptible AKR mice [41]. Nevertheless, mice genetically deficient in eosinophils efficiently produced Th2 cytokines and mediated worm expulsion during primary *T. muris* infection [41]. Moreover, anti-IL-5 neutralization with antibody significantly decreased the production of eosinophils after *T. muris* infection, although the absence of IL-5 and reduced eosinophils had no impact on *T. muris* survival, reproduction and elimination [42,43]. Therefore, it seems likely that eosinophils are not essential for the expulsion of *T. muris* parasites.

##### Mast Cells

The accumulation and activation of innate immune cells, including mast cells at the site of infection in the large intestine, is one of the hallmarks of parasite infections. This is a temporary innate immune response, and these cells return to baseline healthy conditions after the parasite infections have been cleared. However, mast cells still remain accumulated in the site of *T. muris* infection even after several months of worm expulsion [42].

Accumulation of mast cells at the infection site was linked to Th2 immunity activation [9,42] (Figure 1). Thus, mast cell accumulation was not detectable in susceptible mice with dominant Th1 response. Conversely, high dose infection in resistant mice, which promotes Th2 immunity, drives mast cell accumulation [42] and increases worm expulsion. However, there was no impact on *T. muris* expulsion when mast cells were depleted by using a neutralizing antibody for stem cell factor receptor (c-kit receptor) [43]. Since c-kit mutations affect different types of immune cells [25], it was not clear whether other immune cell types apart from mast cells were regulating the expulsion of *T. muris* worms. Hence, the function of mast cells during *T. muris* infection and worm expulsion needs to be further clarified. In addition, it has been indicated that mast-cell-derived proteases control epithelial permeability and consequently maintain barrier integrity [42] (Figure 1). Thus, prior *T. muris* infection may have a long-lasting impact on the gut environment, and these implications on intestinal-barrier integrity might be one of the underlying mechanisms through which mast cells prevent *T. muris* infection in the gut.

##### Natural Killer (NK) Cells

NK cells are innate immune cells that defend the host against both intra- and extracellular (parasites) pathogens. NK cells bind to the Fc portion of antibodies and participate in antibody-dependent cellular cytotoxicity (ADCC) by expressing Fcγ receptors [44]. It was shown that NK cells promote the generation of IL-4 and IL-13 in response to *T. muris* infection, and a delay in worm expulsion was observed in NK cell-deficient mice [45] (Figure 1).

NK cells are one of the important determining factors that mediate the gender-based differences in the immune response against *T. muris* infection [46,47]. *T. muris*-infected female IL-4 knockout (KO) BALB/c mice clear the worms much faster than *T. muris*-infected male IL-4KO mice. NK cells in male IL-4KO mice had higher expression of CXCR3, the chemokine receptor associated with enhanced IFN-γ and Th1 immunity, compared to female counterparts [48,49]. In addition, following *T. muris* infection, the female IL-4KO mice had higher levels of IL-13 produced by DX5^+^ NK cells compared to the male IL-4KO mice [47]. The higher levels of NK cell-derived IL-13 are responsible for the development of resistance against *T. muris* infection in the female IL-4KO mice.

##### Innate Lymphoid Cells (ILCs)

ILCs, non-cytotoxic lymphocytes, are present in lymphoid and non-lymphoid tissues, and they are also abundant at mucosal and non-mucosal barriers [11]. These cells were recently identified and classified into three groups: group 1 ILCs (ILC1s), group 2 ILCs (ILC2s) and group 3 ILCs (ILC3s). Among these groups, ILC2s support Th2-associated responses as well as anti-helminth immunity control [50]. The innate and adaptive immune responses against *T. muris* were believed to be separate mechanisms, but recent research suggests that ILC2s act as a link between these two types of immune responses. During intestinal helminth infections, mature ILC2s produce IL-4, IL-5, IL-9, and IL-13 as well as amphiregulin in response to epithelial cell-derived cytokines such as IL-25, IL-33, and TSLP [51] (Figure 1). The presence of MHC class II on ILC2s facilitates the communication between ILC2s and CD4^+^ T cells to mount Th2 response to helminth infections [52]. The involvement of ILC2s in the secretion of Th2 cytokines reinforces the idea that ILC2s are involved in generating efficient immune responses against *T. muris* and worm expulsion. In contrast, in the inducible costimulatory molecule (iCOS)-T mice, loss of ILC2s caused by diphtheria toxin treatment did not affect *T. muris* expulsion, suggesting ILC2s might not be essential for *T. muris* clearance [53]. Furthermore, a lack of vitamin A led to a significant increase in IL-13-producing ILC2s and *T. muris* resistance; therefore, nutritional stress can be beneficial in response to parasite infections through ILC2 development [54]. This mechanistic finding using the *T. muris* model can partly explain why vitamin A supplementation had no protective effect on soil-transmitted helminthic re-infection [55,56].

#### 2.2.2. Adaptive Immunity

##### T Cells

T cells are important in host protective immunity to many intestinal helminths, including *T. muris.* Transferring T-cell enriched populations (but not B-cell enriched populations) collected from MLN cells of *T. muris*-infected donors to naïve mice with *T. muris* infection conferred immunity and worm expulsion [57]. Nude mice, with congenital absence of thymus, lack T cells and are incapable of generating resistant immune responses. Inoculation of splenocytes or MLN cells or thymocytes from *T. muris-*infected donors restored a resistant phenotype in these nude mice, and their *T. muris* infection was either completely or partially cleared. These studies confirmed the indispensable role of T cells in *T. muris* expulsion [58]. Additionally, *T. muris*-infected severe combined immunodeficiency (SCID) mice failed to expel worms even after receiving CD4^+^ T cells from BALB/c on day 34 p.i., implying that CD4^+^ T cells mediate worm expulsion by affecting *T. muris* larvae [59].

Instead of cytotoxic T cells, protective immune responses against *T. muris* infection are mainly regulated by helper T cells, since using neutralizing antibodies for CD4^+^ T cells but not for CD8^+^ T cells led to the production of susceptible-associated immune responses [60,61,62]. In addition, the adoptive transfer of CD4^+^ T cells from resistant BALB/c mice to the normally susceptible SCID mice resulted in worm elimination. These findings confirm that CD4^+^ T cells, and not CD8^+^ T cells, were critical for generating effective immunity against *T. muris* infection [59]. The ability of CD4^+^ T cells to proliferate and polarize in response to stimuli declines with age, resulting in *T. muris* susceptibility [63]. Among the CD4^+^ T-cell subsets, the Th2 type of immune response is mainly linked with protective immunity in intestinal helminth infection. The key cytokines of the Th2 immune response, IL-4, IL-5, IL-9 and IL-13, are essential in providing immunity against extracellular parasitic infections (Figure 1). Though innate immune cells of the myeloid lineage, namely basophils, eosinophils and mast cells, secrete at least one of these cytokines, CD4^+^ Th2 cells and ILC2s in particular are the major sources of Th2-associated cytokines. It was demonstrated that mice that lacked either IL-4 or IL-13 expression were susceptible to infection with *T. muris* [64,65]. In particular, IL-4 stimulates enterocytes to produce mucin, whereas IL-13 causes goblet cell hyperplasia [11,66] (Figure 1). IL-9 is a pleiotropic cytokine and was primarily studied in the context of Th2-associated immuno-pathological conditions such as parasitic infections. Later studies provided evidence that a distinct subset of CD4+ cells exists that mainly secretes IL-9. These cells were thus termed Th9 cells. These studies suggested that TGF-β, in the presence of IL-4, reprograms CD4^+^ T cells into Th9 cells [67,68]. Along with IL-4 and IL-13, IL-9 is also critical for *T. muris* pathology as evidenced by the inhibition of worm expulsion upon using neutralizing IL-9 antibodies [69]. In addition, the number of CD4^+^ intraepithelial lymphocytes (IELs) in BALB/c mice was increased on day 21 post-infection (close to the peak of expulsion), while IELs in susceptible mice (AKR) were mostly CD8^+^ [31].

##### B Cells

B cells regulate immune responses against *T. muris* infection by acting as APCs, by secreting cytokines and by producing *T. muris*-specific antibodies (Figure 1). In mixed Th1/Th2 conditions, such as in *T. muris*-infected C57BL/6 mice, B cells are required for the enhancement of Th2 responses, while B cells are not essential for the generation of an efficient immune response to *T. muris* in mice with dominant Th2 responses (BALB/c mice) [70]. In support of this, anti-IL-12 antibody treatment in B cell-deficient mice resulted in parasite elimination, as it inhibited the production of the susceptibility-associated Th1 response [71].

Similar to many intestinal parasites, *T. muris* is a macro-pathogen, and therefore macrophages are not able to ingest them. Thus, antibody-dependent cell-mediated cytotoxicity (ADCC) has been identified as a potential alternative method of inducing immune reactions against *Trichuris sp*. Antibodies, such as IgG, IgA, and IgE, produced by B cells coat parasitic antigens, facilitating the process of ADCC [11,62,72] (Figure 1).

Furthermore, resistant and susceptible mice have different types of antibodies. Resistant mice produce high levels of IgG1, a Th2-associated antibody, while IgG2 is increased in susceptible mice with Th1 immune response [71]. Interestingly, an IgA and IgG1 transfer from resistant mice partially restores resistant phenotype in susceptible mice, which is probably because of antibody-induced larval trapping and antibody-mediated neutralization of parasitic antigens [25]. However, [59] demonstrated that *T. muris* antibodies are dispensable for resistance to *T. muris* infection.

##### Regulatory T Cells (Tregs)

Though *T. muris* infection is mainly characterized by Th2 immune response, *T. muris*-infected mice also develop Tregs response. In chronically infected mice, Tregs cells suppress *T. muris*-induced intestinal damage by inhibiting Th2 responses, and therefore, these cells facilitate worm survival. Resistant mice also promote a Tregs response to suppress host pathology induced by Th2 cytokines [73,74]. A prior study investigated the role of Tregs in immune polarization during low-dose *T. muris* infection by using Tregs depletion strategies. Early (during the first 8 days or second 8 days) and late depletion of Tregs in *T. muris*-infected mice (with a low dose of infection) have different immune outcomes. Early Tregs depletion leads to decreased Th1 and enhanced Th2 immune response, and ultimately accelerated *T. muris* expulsion. In contrast, late depletion of Tregs (after infection establishment) increased worm burden in *T. muris-*infected mice. These results confirm that Tregs can restrict Th2 cell expansion during *T. muris* infection before the establishment of T cell polarization [75]. Furthermore, the main cytokine manufactured by Tregs is IL-10. It has been indicated that IL-10-deficient mice with high-dose *T. muris* infection had increased severity of colitis and heavy worm burden, implying IL-10 contributes to the development of Th2 immune response as well as regulation of IFN-γ-induced inflammations [54,76].

## 3. Effects of *T. muris* on the Cells in the Intestinal Epithelial Layer

### 3.1. Effects on Epithelial Cells

Epithelial cells play an important role in *T. muris* infection, notably in the stages of early infection. Mice with deficient nuclear factor-κB (NF- κB) signaling in intestinal epithelial cells fail to generate an effective immune response and expel the worm, showing that epithelial cells need to be stimulated prior to the involvement of the classical immune cells [77]. Intestinal epithelial cells secrete several important cytokines such as IL-25, IL-33 and TSLP that are vital in the development and activation of the Th2 immune response during *T. muris* infection [22] (Figure 1).

IL-25, structurally similar to IL-17, is produced by intestinal epithelial cells that stimulate Th2 immune response to gastrointestinal helminth infections via activation of ILC2s or multipotent progenitor (MPP) cells [22,78,79,80]. Of note, Saenz et al. have eloquently demonstrated how IL-25 develops Th2 responses; IL-25 increases the number of a lineage-negative (Lin2) MPP cells in gut-associated lymphoid tissue. MPP^type2^ cells differentiate into monocyte/macrophage and granulocyte lineage APC cells that in turn promote proliferation and differentiation of T cells to the Th2 phenotype in vivo [80]. Furthermore, IL-25 KO mice are prone to chronic *T. muris* infection with the dominant Th1 cytokines, which can be shifted towards Th2 immunity by MPP^type2^ cell transfer. As a result, MMP^type2^ was identified as a determinant factor in generating resistant immune responses against *T. muris* infection [80].

IL-33 is produced by intestinal epithelial cells and released after injury-mediated cellular necrosis. IL-33 acts biologically by activating NF- κB and MAP kinases via the IL-1 receptor ST2 and has been related to the activation of Th2 immunity in response to helminth infections [22,81]. Compared to susceptible mice, resistant mice have higher levels of IL-33 on day 3 *T. muris* p.i. Furthermore, by giving recombinant IL-33 early in the *T. muris* infection, immune responses in susceptible mice can be reversed. In contrast, late IL-33 treatment does not cause worm expulsions, showing that IL-33 is one of the initial signals during *T. muris* infection that primes Th2 responses. Moreover, IL-33 treatment of *T. muris*-infected SCID mice is not sufficient to produce resistant-associated responses, indicating that the ability of IL-33 to induce Th2 responses is dependent on T cells. In parallel, elevated IL-33 production in resistant mice has been associated with an increase in TSLP (Th2-inducing cytokine) [82].

TSLP is one of the intestinal epithelial cell-derived cytokines that enhances Th2 cell differentiation by acting on a wide range of immune cells, including DCs, basophils, monocytes, granulocytes, T cells, and B cells [22]. In *T. muris* infection, resistant mice have a significant increase in epithelial-derived TSLP level [27]. In addition, during acute *T. muris* infection, TSLP receptor-deficient mice demonstrate the susceptible phenotype [83]. In vitro, TSLP prevents LPS-induced IL-12 synthesis and secretion from murine DCs [77,83,84,85]. Antibody-mediated inhibition of IL-12p40 or IFN-γ restored resistant phenotype in TSLP-deficient mice [83,86]. In addition to DCs, TSLP selectively activates basophil responses through which it can promote Th2 cytokine-mediated responses. It has also been shown that the susceptibility of TSLPR KO mice to *T. muris* infection is associated with a decrease in basophil numbers [34].

Moreover, along with cytokines, chemokines produced by intestinal epithelial cells are equally vital during the pathogenesis of *T. muris* infection. The levels of colonic epithelial chemokines, including CCL2, CCL3, CCL5, and CCL20, are substantially higher in resistant mice than in mice with susceptible infection. Chemokines are effective inducers of DC recruitment, and it has been indicated that giving *T. muris*-resistant mice anti-CCL5 and anti-CCL20 antibodies inhibits DC recruitment in the colon [27].

### 3.2. Effects on Goblet Cells and Mucus Layer

The gastrointestinal epithelium is coated by a viscoelastic mucus layer produced mainly by goblet cells and represents the first line of defense against invading pathogens [87]. Acute and chronic *T. muris* infections result in changes in the components of the intestinal mucus barrier [88]. *T. muris* exposure causes up-regulation of cell surface mucins secretion (*Muc4*, *Muc13* and *Muc17*), which can potentially contribute to the increased thickness of the glycocalyx. Moreover, in acute infection, Th2 cytokine (IL-13) stimulates expression of secretory mucins into the mucus layer via GABA-α3 [88]. This increase in mucins expression and in glycocalyx thickness during acute infection enables the host to clear *T. muris* through worm trapping in mucus, impairing worm motility and inhibiting the feeding capacity of the worm [89].

In the acute *T. muris*-resistant infection model, goblet cell hyperplasia occurs via activation of the transcription factors Math1 and sterile alpha motif (SAM) pointed domain containing ETS transcription factor (SPDEF), which promote the differentiation of stem cells to the secretory cell phenotype [88,90,91]. Conversely, the transcription factor of enterocyte differentiation, Hes-1, had increased expression levels in the chronic *T. muris*-susceptible infection model [88,92]. Goblet cell hyperplasia is believed to be primarily regulated by Th2 cytokines [93,94] (Figure 1), although IL-4/IL-13 independent goblet cell hyperplasia was indicated in some studies [95]. In addition, recently, IL-22, a tissue-protective cytokine, was shown to lead to goblet cell hyperplasia upon *T. muris* infection. It has been shown that *T. muris*-infected IL-22-deficient mice had decreased goblet cell hyperplasia, reduced mucins production (but not *Muc2*), and failed to expel worms [96]. This expansion of goblet cells in mice with acute *T. muris* infection is accompanied by an up-regulation in the secretion of mucins (Figure 1), while during chronic infection, the decreased goblet cell numbers lead to a depleted mucus barrier even though there is hypersecretion of cell surface mucins from the goblet cells [88]. Hasnain and colleagues have shown that *Muc2*, the primary mucin produced by intestinal goblet cells, plays a critical function in the removal of the *T. muris* helminth. Delayed *T. muris* elimination, even with an intact Th2-mediated immune response in *Muc2*-deficient mice, confirmed a distinctive functional role for *Muc2* in host immunity. Along with *Muc2*, *Muc5ac* (a mucin typically expressed in the lungs and stomach) expression was up-regulated during infection shortly before worm clearance in resistant mice [90] (Figure 1). In spite of producing robust Th2 responses, *Muc5ac*-deficient mice were completely unable to expel *T. muris* and harbored long-term, chronic infection, showing the important role of *Muc5ac* in *T. muris* expulsion. Interestingly, the susceptible phenotype in *Muc5ac-*deficient mice, even with anti-IFN-γ administration, did not shift into a Th2-dominated response. Most significantly, human *MUC5AC* has a direct adverse impact on the viability of *T. muris* worms [97].

In addition to quantitative changes in mucus layer components, the quality of mucins is also altered by *T. muris* infection. Chronically infected mice have low-charged mucins, whereas mice with acute *T. muris* infection have highly charged mucins [88]. Furthermore, there is altered glycosylation from sulpho- to sialomucins in the cecum during chronic *T. muris* infection. Sulphomucins, on the other hand, are associated with acute *T. muris* infections and are regulated by IL-13. Notably, sulphate glycan-containing *Muc2* is less vulnerable to proteolytic degradation by *T. muris* ESPs compared to sialomucins [98]. Even with the dominant Th2 immune responses in mice with gene deletion of sulphate anion transporters 1 (Sat-1), susceptible immune responses have been observed to *T. muris* infection due to decreased mucin sulphation [98]. These findings showed that changes in mucin glycosylation induce structural changes in the mucus barrier, which can protect the underlying epithelium from parasite degradation and thereby contribute to host defense.

In addition to mucins, other goblet cell secretory products are also important in the regulation of immune responses against *T. muris* infection. Mice with resistant phenotype express resistin-like molecule-β (RELMβ), which confirms that the expression of RELMβ is not part of a helminth-mediated response, but rather is limited to environments with dominant Th2 protective responses [99,100]. RELMβ production was impaired in mice lacking both IL-4 and IL-13 signals (IL-4Ra KO), but not in the absence of only the IL-4 signal (IL-4 KO mice), implying that IL-13 plays an important role in inducing the RELMβ expression. RELMβ also has a direct inhibitory impact on worms by attaching to helminth chemosensory elements and disrupting the parasitic chemotaxis function in vitro [99]. However, it does not seem to play a role in *T. muris* expulsion, possibly due to the parasite’s active penetration and feeding in the epithelial cells [99,101]. In contrast, studies on chronic *T. muris* infection have demonstrated that RELMβ can enhance Th1 response by triggering intestinal macrophages and subsequently releasing the pro-inflammatory cytokines, namely IL-12/23, IL-6 and TNF-α, and thus promote chronic *T. muris* infection. RELMβ KO mice have reduced T cell-derived IFN-γ and TNF-α and thus decreased *T. muris*-mediated colonic inflammation. Hence, RELMβ-deficient mice are unable to develop chronic infection [101]. In addition, *T. muris* expulsion was linked to the expression of antimicrobial peptides (AMPs) generated by goblet cells (Figure 1), including angiogenin 4 (Ang4) [102,103]. Apart from the production of mucins, RELMβ and AMPs, goblet cells also produce indoleamine 2,3-dioxygenase (IDO), a tryptophan-degrading enzyme that is linked to the pathogenesis of *T. muris* chronic infection. IDO inhibition in *T. muris-*infected SCID mouse increased rates of colonic epithelial cell turnover that resulted in substantial parasite expulsion [103].

### 3.3. Effect on Enteroendocrine Cells

Intestinal enteroendocrine cells (EECs) are specialized epithelial cells, dispersed throughout the gastrointestinal tract that produce various hormones and neuropeptides. EECs have different subsets based on their secretions, including 5-hydroxytryptamine (5-HT) producing enterochromaffin (EC) cells, cholecystokinin (Cck) producing I cells and glucagon-like peptide 1 producing L cells [104].

EECs via its chemosensory function detect helminths and interact with the immune system to orchestrate an immune response [105]. Similar to other epithelial cells, *T. muris* infection drives EEC hyperplasia [106], and specifically, the number of EC cells and 5-HT levels are higher in *T. muris*-infected mice [107] (Figure 1). Our laboratory demonstrated that CD4^+^T cells play an important role in regulation of EC biology in *T. muris* infection [107]. Moreover, we showed that EC cell/5-HT responses in inflammation induced by the *T. muris* are influenced by Th1 or Th2 cytokine predominance, in susceptible and resistant mice, respectively, suggesting the importance of immunological profile in regulation of EC cell biology [108]. In addition, studies from our lab demonstrated that IL-13 mediates the immunological regulation of ECs in response to *T. muris* infection [109].

Our lab has further demonstrated that during *T. muris* infection, the increased synthesis of 5-HT in EC cells is regulated by the gut microbiota via a TLR2-dependent mechanism. The decreased number of EC cells and lower amount of 5-HT in the gut of TLR2-deficient mice and anti-TLR2 antibody-treated mice during acute *T. muris* infection implied that TLR2 is a key innate immune receptor in regulating the response of EC cells to *T. muris* infection [110]. *T. muris* excretory–secretory products (ESPs) induced 5-HT production in BON-1 cells (a model of human EC cells). In addition, the ESPs showed a direct effect on *Tlr2* mRNA and protein expressions, while 5-HT levels were attenuated upon TLR2 antagonist treatment. Therefore, it seems likely that ESPs from *T. muris* can influence EC cell response and 5-HT production via a TLR2 signaling pathway. In addition to influencing EC cells through ESPs, *T. muris* may also indirectly influence 5-HT production via altering the microbial composition. As studies have shown that Th2 cytokines, particularly IL-13, play an important role in 5-HT production from EC cells via the IL-13 receptor, it would be interesting to explore the interaction between TLR2 and IL-13 signaling in future studies.

We have also demonstrated that in the *T. muris* infection model, treatment with peripheral Tph (rate limiting enzyme of 5-HT synthesis) inhibitor and telotristat etiprate (LX1606) enhanced worm expulsion, and increased IL-10 production and goblet cell numbers [111]. However, the precise mechanisms by which 5-HT promote *T. muris* expulsion remain to be determined.

## 4. Effects of *T. muris* on Intestinal Muscle Function

Experimental rodent helminth infection models have been documented to have altered intestinal smooth muscle function. Th2 immune response in the resistant *T. muris* mouse models influences the intestinal microenvironment, including smooth muscle contraction, and thus ultimately the process of worm expulsion [112] (Figure 1). Previously, using the small intestinal helminth *Trichinella spiralis*-infected mice, Vallance et al. showed that the development of intestinal muscle hypercontractility and worm expulsion is highly dependent on CD4^+^ T cells since muscle contraction was attenuated and worm expulsion delayed in athymic CD4-deficient mice [113]. Further investigation into the effects of immunological mechanisms on intestinal muscle function during *T. spiralis* infection revealed that the Th2 cytokines IL-4 and IL-13, but not IL-5, contribute to signal transducer and activator of transcription factor 6 (Stat6)—dependent intestinal muscle hypercontractility and worm expulsion [114,115]. Interestingly, during *T. spiralis,* the infection shift of the immune response from Th2 towards Th1 inhibits infection-induced muscle hypercontractility, prolongs worm survival and delays worm expulsion [116]. Similarly, chronic *T. muris* infection in susceptible AKR mice have a Th1 immune response that is associated with reduced muscle contractility and excitatory innervation. Additionally, after the induction of Th1 immune response, impairment in intestinal muscle function persists even after *T. muris* was expelled [117]. These findings suggest that in countries with endemic parasitic infestations, gut disorders with underlying low-grade inflammation and dysfunctional muscle contractility such as irritable bowel syndrome might be caused by a Th1-biased chronic parasitic infection [117].

The dissimilarities in immune-mediated muscle response in distinct helminth infections came into sight when our lab showed that another cytokine IL-9, which is not a Stat6 activator, regulates intestinal muscle function in *T. spiralis* and *T. muris* infection rather differently [69] (Figure 1). IL-9 treatment enhanced jejunal muscle contraction and augmented worm expulsion in *T. spiralis* infection. There were also increases in IL-4 and IL-13 production from in vitro stimulated spleen cells isolated from IL-9-treated mice. However, endogenous IL-9 is not essential for intestinal muscle contraction in *T. spiralis*-infected mice since removal of IL-9 by anti-IL-9 vaccination or by anti-IL-9 antibody had no effect on worm expulsion or muscle contraction. From these observations, it can be hypothesized that IL-9 administration increases hypercontractility in *T. spiralis* infection either by its additive effect with other Th2 cytokines or by increasing the levels of IL-4 and IL-13. In contrast, neutralization of IL-9 by anti-IL-9 vaccination significantly reduced colonic muscle hypercontractility and suppressed worm expulsion in *T. muris* infection [69]. Our findings were further supported by the observation of faster worm expulsion in IL-9 overexpressing transgenic *T. muris-*infected mice compared to wild-type mice [118]. Thus, it can be affirmed that in regard to intestinal muscle contraction, while IL-9 is vital in the development of increased colonic muscle contraction in *T. muris* infection, it is not essential in *T. spiralis* infection [69]. In addition, [119] also demonstrated that IL-33-ST2 signaling stimulates EC cells to synthesize and secrete 5-HT, which activates enteric neurons and promotes gut motility, resulting in *T. muris* expulsion.

Another school of thought is that the expulsion of worms is also dependent on intestinal epithelial cell proliferation and turnover in addition to peristalsis. Increased epithelial proliferation and rapid turnover shift the epithelium away from the crypts and facilitates *T. muris* expulsion from within their epithelial niche [25,106]. In addition, increased intestinal muscle contraction could probably help in shedding the *T. muris-*infected epithelial cells [25]. Thus, it is evident that *T. muris* infection elicits an immune response that regulates intestinal smooth muscle function and worm expulsion in concert with other mechanisms that are yet to be explored.

## 5. Interaction of *T. muris* with Gut Microbiota

In healthy conditions, complex and diverse gut bacterial communities affect intestinal physiology and immunity. During parasite infections, gut microbiota share the gut ecosystem with intestinal parasites, with both having an impact on the host physiology and immune landscape. This proximity between microbiota and intestinal parasites gives them a chance to interact. These parasite–microbiota interactions can shift host immune responses against gut microbiota towards either pro-inflammatory or anti-inflammatory responses. On the other hand, the gut microbiota affect colonization, reproduction and infectivity of parasites and change parasitism towards mutualism. Contemporary methods such as 16s sequencing have shed light on the parasites–microbiota interactions and how these interactions affect the host health [120,121]. Gut microbiota can have direct effects on the life cycle of *T. muris*, by attaching to polar caps and facilitating egg hatching and ultimately favoring the establishment of *T. muris* infection [122]. *T. muris* itself alters the microbiota composition towards a less favorable environment in the gut that impedes further *T. muris* establishment. Thus, these microbiota facilitate the development of chronic infection by inhibiting egg hatching of a second dose of infection and controlling numbers of established *T. muris* infection [123]. Furthermore, it was recently discovered that *T. muris* selects and acquires some specific intestinal bacterial subsets from the gut environment of the murine host that facilitate its survival [123]. In addition, [124] showed that relocation of C57BL/6 mice, from a laboratory to a farm-like environment, causes changes in microbiota composition and diversity that contribute to the microbial enhancement of Th1 immunity and increase *T. muris* susceptibility. These findings confirmed that *T. muris* survival is immensely influenced by host microbiota and these microbiota may have a role in immune responses generated against *T. muris* infection. Further studies are required to elucidate the role of *T. muris*-induced altered gut microbiota in regulating anti-helminth immune responses.

*T. muris* can also change the physical environment of the intestine and impact the habitat of gut microbiota [121]. *T. muris* infection can have consequences on some subsets of the gut microbiota survival by imposing physical changes in the mucus layer by altering the structure and quantity of mucins being produced [90,97,121]. *T. muris*-induced physical changes in the mucus layer alter the accessibility of intestinal microbiota to nutrients and impair the attachment of microbiota to the intestinal epithelium. Studies showed that *T. muris* infection is linked to abundant *Mucispirillum* [125,126] and *Clostridiales* in the gut. In vitro findings of accelerated growth of *Clostridiales* strains in a mucin-rich environment confirmed that *T. muris*-mediated changes in the mucus layer are beneficial to *Clostridiales* [127].

Chronic *T. muris* infection in IL-10-deficient mice raises *Lactobacillaceae* populations. Conversely, in acute *T. muris* infection, the cecal microbial diversity did not change significantly [128]. These findings suggest that the dominant immune response generated against chronic or resistant *T. muris* infection and changes in the intestinal microenvironment may play an important role in altering the host microbiota. However, no studies have explained the consequences of microbiota-induced gut physical changes on *T. muris* infection outcomes [120].

Large intestinal parasites may also alter the structure and composition of the gut microbiota indirectly by regulating the synthesis and secretion of antimicrobial peptides (AMPs) by the host goblet cells. *T. muris* increases the expression of the AMP, Ang4 from goblet cells, in resistant mice, and it was shown that elevated Ang4 expression is linked with increased *T. muris* expulsion [129]. However, the mechanism by which *T. muris*-induced increased Ang4 expression contributes to worm clearance is not very clear. Reportedly, Ang4 promotes the clearance of worms by either having a direct toxic effect on enteric parasites [130] or by altering the microbial composition and ultimately affecting the host immune response to *T. muris* [121].

Furthermore, the microbial changes during *T. muris* infection contribute to intestinal pathology such as colitis. Using Nod2 KO mice that develop spontaneous colitis, it was observed that *T. muris* infection induces Th2 immunity that leads to the expansion of the beneficial bacteria *Clostridiales* that competitively inhibits colitogenic *Bacteroides vulgatus* and ultimately decreases the severity of colitis [127]. These findings showed that study of the bidirectional relationships between *T. muris* and microbiota is vital for understanding gut pathology and employing these interactions as a therapy for gastrointestinal disorders.

## 6. Role of *T. muris* in the Modulation of Immune and Inflammatory Disorders

**Figure 2:**
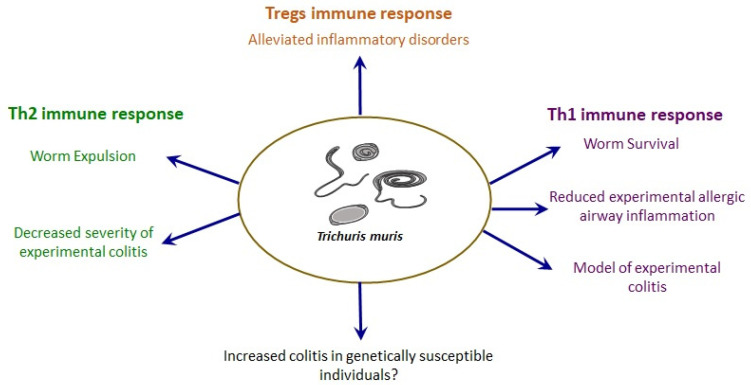
Overview of the role of *T. muris* in inflammatory and immunogenic diseases. *T. muris* modulates immunopathological and inflammatory disorders by triggering Th1/Th2/Tregs immune responses [9]. Acute *T. muris* infection results in mounting Th2 immune responses, which are associated with worm expulsion and reduced severity of chemical-induced colitis [135]. However, the effect of *T. muris* infection in colitis in genetically susceptible individuals remains to be determined. Chronic *T. muris* infection leads to Th1 immune responses resulting in worm survival and protection against allergic airway diseases [146]. Chronic *T. muris* infection model can also be used as a model of experimental colitis [144]. *T. muris* infection also induces regulatory Tregs, and consequently these cells, by releasing anti-inflammatory cytokines, reduce inflammatory disorders [141].

The incidence of autoimmune, allergic and inflammatory diseases is on the rise in industrialized societies, where infectious diseases such as helminth infections have decreased [131]. Using this rationale, the proposed “hygiene hypothesis” suggests that the rising incidence of immunopathological disorders may partially be due to increased hygiene, sanitation and de-worming strategies [131,132]. The hygiene hypothesis is supported by studies with the pig whipworm *T.* *suis*, where *T. suis* was seen to alleviate the severity of inflammation in inflammatory bowel disease (IBD) patients, even though the sample size was relatively small [133,134]. In a similar thread, Vegas-Sanchez et al. observed that the simultaneous induction of dextran sulphate sodium-induced colitis and *T. muris* infection in mice results in improvement of colitis due to the host’s tolerance *to T. muris* [135]. This makes *T. muris* an appropriate model to study the beneficial effects of host–parasite immune interactions in modulating IBD. In Th1-mediated Crohn’s Disease (CD), a type of IBD, it is proposed that helminthic infections such as *T. muris* can activate the production of Th2 cytokines that can antagonize the disease-promoting Th1 environment in the gut [134] (Figure 2). Studies demonstrated that other helminths such as *Heligomosomoides polygyrus*, *Schistosoma mansoni, Hymenolepis diminuta* and *T. spiralis* shift the immune response towards Th2 production and reduce the severity of colitis [136,137,138,139]. Our laboratory also demonstrated that previous treatment with *T. spiralis* antigens in mice decreased the intensity of colitis and the mortality rate significantly [140]. Infection by helminth parasites also induces regulatory Tregs, and these cells consequently secrete regulatory cytokines such as IL-10 and TGF-β. These regulatory cells and their anti-inflammatory cytokines may also contribute to beneficial effects of helminth in inflammatory disorders [141] (Figure 2). However, in the genetically susceptible *Mdr1a^−/−^* (epithelial transporter gene) mice model, which develops spontaneous colitis, infection with *T. muris* augmented the progression of colitis indicated by a significant increase in histological damage score, pro-inflammatory cytokines, and infiltration of mucosal CD4+ T-cell and DCs [142] (Figure 2). Similarly, Wilson and colleagues revealed the development of a lethal colitis in *Il-10^−/−^* mice infected with *T. muris,* though the amount to which the parasite enhanced the colitis is not clear [143]. The conflicting results seen with *T. muris* infection and colitis severity might be explained by the difference in colitis models, stage of colitis, persistence and burden of worms. Furthermore, *T. muris* chronic infection in the susceptible AKR mice model shows marked phenotypic and transcriptional similarities to experimental models of IBD with the greatest similarity to the T-cell transfer model of colitis [144]. *T. muris-*infected AKR mice had up-regulated expression of genes involved in innate and adaptive immune response, chemotaxis and apoptosis with the predominant activity of the Th1/Th17 pathway [144]. These findings validate *T. muris* infection as a model of experimental colitis as well as a model for determination of factors that promote development and severity of chronic colitis [111,144,145] (Figure 2).

Central to the concept of the hygiene hypothesis is the maintenance of the appropriate balance between the Th1 and Th2 type immune responses in a healthy state. Previously, based on this hypothesis, it was suggested that reduced childhood bacterial and viral infections result in inadequate Th1 responses, which in turn cannot compensate the expansion of Th2 cells and therefore increase the predisposition to allergy. However, this hypothesis with respect to allergy was brought into question due to the observation that helminthic infections, one of the most potent Th2 response stimuli, are inversely correlated with allergy [134,147]. The dose, chronicity and timing of helminthic infections are important factors that determine whether the infection is harmful or protective for allergic disease development [147]. Using the *T. muris* model, Chenery et al. showed that low-dose *T. muris* intestinal infection in C57BL/6J mice leads to the production of Th1 cell-dependent IFN-γ and myeloid cell-derived IL-10 in the lung without any airway pathology. This immune response resulted in the inhibition of papain-induced acute allergic airway inflammation (Figure 2). During *T. muris* infection, IFN-γ from the Th1 cells might have inhibited the Th2 immune response in allergic airway inflammation. Moreover, the group also observed that low dose *T. muris* infection is protective against the house dust-mite model of murine asthma only when the mice were infected with *T. muris* prior to sensitization [146] (Figure 2). Helminth therapy with *T. suis* in relapsing remitting multiple sclerosis (MS) patients was shown to increase serum IL-4 and IL-10 and decrease in disease severity with no serious adverse events of the helminth infection [148]. In the animal model of rheumatoid arthritis (RA), helminth therapy with *S. mansoni* and *S. japonicum* showed attenuation of disease severity via up-regulation of IL-4 and IL-10 [149]. Collectively, these findings suggest a potential beneficial role for “helminthic therapy” in allergic diseases, IBD and autoimmune diseases such as MS and RA. Furthermore, it is evident from various studies that the *T. muris* infection model in rodents is an extremely useful and convenient tool in teasing out the underlying complex immunological mechanisms of inflammatory, autoimmune and allergic diseases.

## 7. Conclusions

The *T. muris* model has been used to uncover reciprocal host–parasite interactions, host defense, inflammation and protective immune mechanisms. The *T. muris* life cycle and immune response generated against *T. muris* infection are affected by various factors, including host genetic background, host strain, gender, dose of infection and *T. muris* worm strains. A wide range of innate immune cells responds to *T. muris* infection via presenting Ags to adaptive immune cells, participating in ADCC and by producing cytokines. Adaptive immune cells, by producing Th2 cytokines, are key players in generating a resistant immune response to *T. muris* infection. Th2 cytokines contribute to worm expulsion by accelerating epithelial turnover, and increasing mucin secretion and intestinal muscle contractility.

*T. muris* completes its life cycle mainly in the large intestine in close contact with the intestinal epithelium. Thus, mucus layer, enterocytes and specialized epithelial cells act as the first line of defense to restrict *T. muris* infection and invasion prior to the recruitment of classical immune cells to the site of infection. Among epithelial cells, goblet cells are one of the key players essential for *T. muris* clearance by producing the mucins *Muc2* and *Muc5ac*. However, some immunological mechanisms in response to *T. muris* infection are not yet understood. In particular, it remains unclear how *T. muris* infection initiates immune responses and which immune factors are involved in the early stages of *T muris* infection. Furthermore, the gut microbiota are another critical aspect in the study of immune responses in the *T. muris* model. The complex interactions between gut microbiota and *T. muris* influence the survival of *T. muris* in the host and also change the microbial composition. Due to limited study in the field of parasite–microbiota interactions, it is not very clear whether the altered microbiota play a role in the response of the immune system to *T. muris* infection. Considering the recent demonstration of the influence of 5-HT in modulation of gut microbial composition [150], it will be also interesting to explore whether *T. muris*-induced changes in gut microbiota can contribute to host defense in this infection.

Based on the principles of “hygiene hypothesis”, *T. muris* infection can be beneficial in protecting the host from autoimmune, allergic and inflammatory diseases. It has been suggested that *T. muris* infection may alleviate Th1-mediated inflammation such as CD by activating Th2 responses [131,132]. Using the chronic *T. muris* model, it was seen that the Th1 immune response was also beneficial for counteracting the Th2-mediated airway allergic inflammation [146]. These promising findings indicate the potential for the *T. muris* model to further understand the pathological mechanisms of complex immunological disorders and also as a future candidate of helminth therapy for immune and inflammatory diseases.

## Data Availability

Not applicable.

## References

[1] Wright J.E., Werkman M., Dunn J.C., Anderson R.M. (2018). Current epidemiological evidence for predisposition to high or low intensity human helminth infection: A systematic review. Parasit. Vectors.

[2] Dunn J.C., Turner H.C., Tun A., Anderson R.M. (2016). Epidemiological surveys of, and research on, soil-transmitted helminths in Southeast Asia: A systematic review. Parasit. Vectors.

[3] Hotez P.J., Fenwick A., Savioli L., Molyneux D.H. (2009). Rescuing the bottom billion through control of neglected tropical diseases. Lancet.

[4] Cruz K., Marcilla A., Kelly P., Vandenplas M., Osuna A., Trelis M. (2021). *Trichuris trichiura* egg extract proteome reveals potential diagnostic targets and immunomodulators. PLoS Negl. Trop. Dis..

[5] Gilman R.H., Chong Y.H., Davis C., Greenberg B., Virik H.K., Dixon H.B. (1983). The adverse consequences of heavy Trichuris infection. Trans. R. Soc. Trop. Med. Hyg..

[6] World Health Organization (2005). Deworming for Health and Development: Report of the Third Global Meeting of the Partners for Parasite Control.

[7] Zaph C., Cooper P.J., Harris N.L. (2014). Mucosal immune responses following intestinal nematode infection. Parasite Immunol..

[8] Khan W.I. (2008). Physiological changes in the gastrointestinal tract and host protective immunity: Learning from the mouse-*Trichinella spiralis* model. Parasitology.

[9] Klementowicz J.E., Travis M.A., Grencis R.K. (2012). *Trichuris muris*: A model of gastrointestinal parasite infection. In Proceedings of the Seminars in immunopathology. Semin. Immunopathol..

[10] Cliffe L.J., Grencis R.K. (2004). The *Trichuris muris* system: A paradigm of resistance and susceptibility to intestinal nematode infection. Adv. Parasitol..

[11] Darlan D.M., Rozi M.F., Yulfi H. (2021). Overview of Immunological Responses and Immunomodulation Properties of Trichuris sp.: Prospects for Better Understanding Human Trichuriasis. Life.

[12] Tilney L.G., Connelly P.S., Guild G.M., Vranich K.A., Artis D. (2005). Adaptation of a nematode parasite to living within the mammalian epithelium. J. Exp. Zool. Part A Comp. Exp. Biol..

[13] Hansen T.V.A., Hansen M., Nejsum P., Mejer H., Denwood M., Thamsborg S.M. (2016). Glucose absorption by the bacillary band of *Trichuris muris*. PLoS Negl. Trop. Dis..

[14] Else K.J., Wakelin D., Wassom D.L., Hauda K.M. (1990). The influence of genes mapping within the major histocompatibility complex on resistance to *Trichuris muris* infections in mice. Parasitology.

[15] Else K., Wakelin D. (1988). The effects of H-2 and non-H-2 genes on the expulsion of the nematode *Trichuris muris* from inbred and congenic mice. Parasitology.

[16] Schwartz R.H. (1985). T-lymphocyte recognition of antigen in association with gene products of the major histocompatibility complex. Annu. Rev. Immunol..

[17] Hepworth M.R., Hardman M.J., Grencis R.K. (2010). The role of sex hormones in the development of Th2 immunity in a gender-biased model of *Trichuris muris* infection. Eur. J. Immunol..

[18] Bancroft A.J., Else K.J., Grencis R.K. (1994). Low level infection with *Trichuris muris* significantly affects the polarization of the CD4 response. Eur. J. Immunol..

[19] Bancroft A.J., Else K.J., Humphreys N.E., Grencis R.K. (2001). The effect of challenge and trickle *Trichuris muris* infections on the polarisation of the immune response. Int. J. Parasitol..

[20] Koyama K., Ito Y. (1996). Comparative studies on immune responses to infection in susceptible B10. BR mice infected with different strains of the murine nematode parasite *Trichuris muris*. Parasite Immunol..

[21] Bellaby T., Robinson K., Wakelin D. (1996). Induction of differential T-helper-cell responses in mice infected with variants of the parasitic nematode *Trichuris muris*. Infect. Immun..

[22] Harris N.L. (2017). Recent advances in type-2-cell-mediated immunity: Insights from helminth infection. Immunity.

[23] Coakley G., Harris N.L. (2020). Interactions between macrophages and helminths. Parasite Immunol..

[24] Little M.C., Hurst R.J.M., Else K.J. (2014). Dynamic changes in macrophage activation and proliferation during the development and resolution of intestinal inflammation. J. Immunol..

[25] Sorobetea D., Svensson-Frej M., Grencis R. (2018). Immunity to gastrointestinal nematode infections. Mucosal Immunol..

[26] De Schoolmeester M.L., Martinez Pomares L., Gordon S., Else K.J. (2009). The mannose receptor binds *Trichuris muris* excretory/secretory proteins but is not essential for protective immunity. Immunology.

[27] Cruickshank S.M., De Schoolmeester M.L., Svensson M., Howell G., Bazakou A., Logunova L., Little M.C., English N., Mack M., Grencis R.K. (2009). Rapid dendritic cell mobilization to the large intestinal epithelium is associated with resistance to *Trichuris muris* infection. J. Immunol..

[28] Mayer J.U., Demiri M., Agace W.W., MacDonald A.S., Svensson-Frej M., Milling S.W. (2017). Different populations of CD11b+ dendritic cells drive Th2 responses in the small intestine and colon. Nat. Commun..

[29] Joeris T., Müller-Luda K., Agace W.W., Mowat A.M. (2017). Diversity and functions of intestinal mononuclear phagocytes. Mucosal Immunol..

[30] Demiri M., Müller-Luda K., Agace W.W., Svensson-Frej M. (2017). Distinct DC subsets regulate adaptive Th1 and 2 responses during *Trichuris muris* infection. Parasite Immunol..

[31] Little M.C., Bell L.V., Cliffe L.J., Else K.J. (2005). The characterization of intraepithelial lymphocytes, lamina propria leukocytes, and isolated lymphoid follicles in the large intestine of mice infected with the intestinal nematode parasite *Trichuris muris*. J. Immunol..

[32] Else K.J., Finkelman F.D., Maliszewski C.R., Grencis R.K. (1994). Cytokine-mediated regulation of chronic intestinal helminth infection. J. Exp. Med..

[33] Webb L.M., Wojno E.D.T. (2017). The role of rare innate immune cells in Type 2 immune activation against parasitic helminths. Parasitology.

[34] Siracusa M.C., Saenz S.A., Hill D.A., Kim B.S., Headley M.B., Doering T.A., Wherry E.J., Jessup H.K., Siegel L.A., Kambayashi T. (2011). TSLP promotes interleukin-3-independent basophil haematopoiesis and type 2 inflammation. Nature.

[35] Perrigoue J.G., Saenz S.A., Siracusa M.C., Allenspach E.J., Taylor B.C., Giacomin P.R., Nair M.G., Du Y., Zaph C., Van Rooijen N. (2009). MHC class II–dependent basophil–CD4+ T cell interactions promote TH2 cytokine–dependent immunity. Nat. Immunol..

[36] Webb L.M., Oyesola O.O., Früh S.P., Kamynina E., Still K.M., Patel R.K., Peng S.A., Cubitt R.L., Grimson A., Grenier J.K. (2019). The Notch signaling pathway promotes basophil responses during helminth-induced type 2 inflammation. J. Exp. Med..

[37] Kim S., Prout M., Ramshaw H., Lopez A.F., LeGros G., Min B. (2010). Cutting edge: Basophils are transiently recruited into the draining lymph nodes during helminth infection via IL-3, but infection-induced Th2 immunity can develop without basophil lymph node recruitment or IL-3. J. Immunol..

[38] Phythian-Adams A.T., Cook P.C., Lundie R.J., Jones L.H., Smith K.A., Barr T.A., Hochweller K., Anderton S.M., Hämmerling G.J., Maizels R.M. (2010). CD11c depletion severely disrupts Th2 induction and development in vivo. J. Exp. Med..

[39] Sullivan B.M., Liang H.-E., Bando J.K., Wu D., Cheng L.E., McKerrow J.K., Allen C.D.C., Locksley R.M. (2011). Genetic analysis of basophil function in vivo. Nat. Immunol..

[40] Dixon H., Blanchard C., Deschoolmeester M.L., Yuill N.C., Christie J.W., Rothenberg M.E., Else K.J. (2006). The role of Th2 cytokines, chemokines and parasite products in eosinophil recruitment to the gastrointestinal mucosa during helminth infection. Eur. J. Immunol..

[41] Svensson M., Bell L., Little M.C., De Schoolmeester M., Locksley R.M., Else K.J. (2011). Accumulation of eosinophils in intestine draining mesenteric lymph nodes occurs after *Trichuris* muris infection. Parasite Immunol..

[42] Sorobetea D., Holm J.B., Henningsson H., Kristiansen K., Svensson Frej M. (2017). Acute infection with the intestinal parasite *Trichuris muris* has long term consequences on mucosal mast cell homeostasis and epithelial integrity. Eur. J. Immunol..

[43] Betts C.J., Else K.J. (1999). Mast cells, eosinophils and antibody mediated cellular cytotoxicity are not critical in resistance to *Trichuris muris*. Parasite Immunol..

[44] Brillantes M., Beaulieu A.M. (2020). Memory and memory-like NK cell responses to microbial pathogens. Front. Cell. Infect. Microbiol..

[45] Krauss M.E.Z. (2018). CD4+ T Cell Metabolism during Trichuris muris Infection.

[46] Hayes K.S., Bancroft A.J., Grencis R.K. (2007). The role of TNF-α in *Trichuris muris* infection I: Influence of TNF-α receptor usage, gender and IL-13. Parasite Immunol..

[47] Hepworth M.R., Grencis R.K. (2009). Disruption of Th2 immunity results in a gender-specific expansion of IL-13 producing accessory NK cells during helminth infection. J. Immunol..

[48] Martín-Fontecha A., Thomsen L.L., Brett S., Gerard C., Lipp M., Lanzavecchia A., Sallusto F. (2004). Induced recruitment of NK cells to lymph nodes provides IFN-γ for TH 1 priming. Nature immunology. Nat. Immunol..

[49] Wald O., Weiss I.D., Wald H., Shoham H., Bar-Shavit Y., Beider K., Galun E., Weiss L., Flaishon L., Shachar I. (2006). IFN-γ acts on T cells to induce NK cell mobilization and accumulation in target organs. J. Immunol..

[50] Artis D., Spits H. (2015). The biology of innate lymphoid cells. Nature.

[51] Kumar V. (2014). Innate lymphoid cells: New paradigm in immunology of inflammation. Immunol. Lett..

[52] Oliphant C.J., Hwang Y.Y., Walker J.A., Salimi M., Wong S.H., Brewer J.M., Englezakis A., Barlow J.L., Hams E., Scanlon S.T. (2014). MHCII-mediated dialog between group 2 innate lymphoid cells and CD4+ T cells potentiates type 2 immunity and promotes parasitic helminth expulsion. Immunity.

[53] Glover M., Colombo S.A.P., Thornton D.J., Grencis R.K. (2019). Trickle infection and immunity to *Trichuris muris*. PLoS Pathog..

[54] Grencis R.K., Humphreys N.E., Bancroft A.J. (2014). Immunity to gastrointestinal nematodes: Mechanisms and myths. Immunol. Rev..

[55] Isah A.U.J., Ekwunife O.I., Ejie I.L., Mandrik O. (2020). Effects of nutritional supplements on the re-infection rate of soil-transmitted helminths in school-age children: A systematic review and meta-analysis. PLoS ONE.

[56] Al-Mekhlafi H.M., Anuar T.S., Al-Zabedi E.M., Al-Maktari M.T., Mahdy M.A.K., Ahmed A., Sallam A.A., Abdullah W.A., Moktar N., Surin J. (2014). Does vitamin A supplementation protect schoolchildren from acquiring soil-transmitted helminthiasis? A randomized controlled trial. Parasit. Vectors.

[57] Lee T.D.G., Wakelin D., Grencis R.K. (1983). Cellular mechanisms of immunity to the nematode *Trichuris muris*. Int. J. Parasitol..

[58] Yoichi I. (1991). The absence of resistance in congenitally athymic nude mice toward infection with the intestinal nematode, *Trichuris muris*: Resistance restored by lymphoid cell transfer. Int. J. Parasitol..

[59] Else K.J., Grencis R.K. (1996). Antibody-independent effector mechanisms in resistance to the intestinal nematode parasite *Trichuris muris*. Infect. Immun..

[60] Koyama K., Tamauchi H., Ito Y. (1995). The role of CD4+ and CD8+ T cells in protective immunity to the murine nematode parasite *Trichuris muris*. Parasite Immunol..

[61] Humphreys N.E., Worthington J.J., Little M.C., Rice E.J., Grencis R.K. (2004). The role of CD8+ cells in the establishment and maintenance of a *Trichuris muris* infection. Parasite Immunol..

[62] Koyama K. (2002). NK1. 1+ cell depletion in vivo fails to prevent protection against infection with the murine nematode parasite *Trichuris muris*. Parasite Immunol..

[63] Humphreys N.E., Grencis R.K. (2002). Effects of ageing on the immunoregulation of parasitic infection. Infect. Immun..

[64] Bancroft A.J., McKenzie A.N.J., Grencis R.K. (1998). A critical role for IL-13 in resistance to intestinal nematode infection. J. Immunol..

[65] Urban J.F., Madden K.B., Svetica A., Cheever A., Trotta P.P., Gause W.C., Katona I.M., Finkelman F.D. (1992). The importance of Th2 cytokines in protective immunity to nematodes. Immunol. Rev..

[66] Sharba S., Navabi N., Padra M., Persson J.A., Quintana-Hayashi M.P., Gustafsson J.K., Szeponik L., Venkatakrishnan V., Sjöling Å., Nilsson S. (2019). Interleukin 4 induces rapid mucin transport, increases mucus thickness and quality and decreases colitis and Citrobacter rodentium in contact with epithelial cells. Virulence.

[67] Dardalhon V., Awasthi A., Kwon H., Galileos G., Gao W., Sobel R.A., Mitsdoerffer M., Strom T.B., Elyaman W., Ho I.-C. (2008). IL-4 inhibits TGF-β-induced Foxp3+ T cells and, together with TGF-β, generates IL-9+ IL-10+ Foxp3− effector T cells. Nat. Immunol..

[68] Veldhoen M., Uyttenhove C., Van Snick J., Helmby H., Westendorf A., Buer J., Martin B., Wilhelm C., Stockinger B. (2008). Transforming growth factor-β’reprograms’ the differentiation of T helper 2 cells and promotes an interleukin 9–producing subset. Nat. Immunol..

[69] Khan W.I., Richard M., Akiho H., Blennerhasset P.A., Humphreys N.E., Grencis R.K., Van Snick J., Collins S.M. (2003). Modulation of intestinal muscle contraction by interleukin-9 (IL-9) or IL-9 neutralization: Correlation with worm expulsion in murine nematode infections. Infect. Immun..

[70] Sahputra R., Ruckerl D., Couper K.N., Muller W., Else K.J. (2019). The essential role played by B cells in supporting protective immunity against *Trichuris muris* infection is by controlling the Th1/Th2 balance in the mesenteric lymph nodes and depends on host genetic background. Front. Immunol..

[71] Blackwell N.M., Else K.J. (2001). B cells and antibodies are required for resistance to the parasitic gastrointestinal nematode *Trichuris muris*. Infect. Immun..

[72] Makepeace B.L., Martin C., Turner J.D., Specht S. (2012). Granulocytes in helminth infection-who is calling the shots?. Curr. Med. Chem..

[73] D’Elia R., Behnke J.M., Bradley J.E., Else K.J. (2009). Regulatory T cells: A role in the control of helminth-driven intestinal pathology and worm survival. J. Immunol..

[74] Rausch S., Huehn J., Loddenkemper C., Hepworth M.R., Klotz C., Sparwasser T., Hamann A., Lucius R., Hartmann S. (2009). Establishment of nematode infection despite increased Th2 responses and immunopathology after selective depletion of Foxp3+ cells. Eur. J. Immunol..

[75] Sawant D.V., Gravano D.M., Vogel P., Giacomin P., Artis D., Vignali D.A.A. (2014). Regulatory T cells limit induction of protective immunity and promote immune pathology following intestinal helminth infection. J. Immunol..

[76] Schopf L.R., Hoffmann K.F., Cheever A.W., Urban J.F., Wynn T.A. (2002). IL-10 is critical for host resistance and survival during gastrointestinal helminth infection. J. Immunol..

[77] Zaph C., Troy A.E., Taylor B.C., Berman-Booty L.D., Guild K.J., Du Y., Yost E.A., Gruber A.D., May M.J., Greten F.R. (2007). Epithelial-cell-intrinsic IKK-β expression regulates intestinal immune homeostasis. Nature.

[78] Owyang A.M., Zaph C., Wilson E.H., Guild K.J., McClanahan T., Miller H.R.P., Cua D.J., Goldschmidt M., Hunter C.A., Kastelein R.A. (2006). Interleukin 25 regulates type 2 cytokine-dependent immunity and limits chronic inflammation in the gastrointestinal tract. J. Exp. Med..

[79] Fallon P.G., Ballantyne S.J., Mangan N.E., Barlow J.L., Dasvarma A., Hewett D.R., McIlgorm A., Jolin H.E., McKenzie A.N.J. (2006). Identification of an interleukin (IL)-25–dependent cell population that provides IL-4, IL-5, and IL-13 at the onset of helminth expulsion. J. Exp. Med..

[80] Saenz S.A., Siracusa M.C., Perrigoue J.G., Spencer S.P., Urban J.F., Tocker J.E., Budelsky A.L., Kleinschek M.A., Kastelein R.A., Kambayashi T. (2010). IL25 elicits a multipotent progenitor cell population that promotes TH 2 cytokine responses. Nature.

[81] Schmitz J., Owyang A., Oldham E., Song Y., Murphy E., McClanahan T.K., Zurawski G., Moshrefi M., Qin J., Li X. (2005). IL-33, an interleukin-1-like cytokine that signals via the IL-1 receptor-related protein ST2 and induces T helper type 2-associated cytokines. Immunity.

[82] Humphreys N.E., Xu D., Hepworth M.R., Liew F.Y., Grencis R.K. (2008). IL-33, a potent inducer of adaptive immunity to intestinal nematodes. J. Immunol..

[83] Taylor B.C., Zaph C., Troy A.E., Du Y., Guild K.J., Comeau M.R., Artis D. (2009). TSLP regulates intestinal immunity and inflammation in mouse models of helminth infection and colitis. J. Exp. Med..

[84] Liu Y.-J., Soumelis V., Watanabe N., Ito T., Wang Y.-H., de Waal Malefyt R., Omori M., Zhou B., Ziegler S.F. (2007). TSLP: An epithelial cell cytokine that regulates T cell differentiation by conditioning dendritic cell maturation. Annu. Rev. Immunol..

[85] Soumelis V., Reche P.A., Kanzler H., Yuan W., Edward G., Homey B., Gilliet M., Ho S., Antonenko S., Lauerma A. (2002). Human epithelial cells trigger dendritic cell–mediated allergic inflammation by producing TSLP. Nat. Immunol..

[86] Massacand J.C., Stettler R.C., Meier R., Humphreys N.E., Grencis R.K., Marsland B.J., Harris N.L. (2009). Helminth products bypass the need for TSLP in Th2 immune responses by directly modulating dendritic cell function. Proc. Natl. Acad. Sci. USA.

[87] Dharmani P., Srivastava V., Kissoon-Singh V., Chadee K. (2009). Role of intestinal mucins in innate host defense mechanisms against pathogens. J. Innate Immun..

[88] Hasnain S.Z., Thornton D.J., Grencis R.K. (2011). Changes in the mucosal barrier during acute and chronic *Trichuris muris* infection. Parasite Immunol..

[89] Kim J.J., Khan W.I. (2013). Goblet cells and mucins: Role in innate defense in enteric infections. Pathogens.

[90] Hasnain S.Z., Wang H., Ghia J., Haq N., Deng Y., Velcich A., Grencis R.K., Thornton D.J., Khan W.I. (2010). Mucin gene deficiency in mice impairs host resistance to an enteric parasitic infection. Gastroenterology.

[91] Else K.J. (2005). Have gastrointestinal nematodes outwitted the immune system?. Parasite Immunol..

[92] Artis D., Potten C.S., Else K.J., Finkelman F.D., Grencis R.K. (1999). *Trichuris muris*: Host intestinal epithelial cell hyperproliferation during chronic infection is regulated by interferon-γ. Exp. Parasitol..

[93] McKenzie G.J., Bancroft A., Grencis R.K., McKenzie A.N.J. (1998). A distinct role for interleukin-13 in Th2-cell-mediated immune responses. Curr. Biol..

[94] Khan W.I., Blennerhasset P., Ma C., Matthaei K.I., Collins S.M. (2001). Stat6 dependent goblet cell hyperplasia during intestinal nematode infection. Parasite Immunol..

[95] Marillier R.G., Michels C., Smith E.M., Fick L.C.E., Leeto M., Dewals B., Horsnell W.G.C., Brombacher F. (2008). IL-4/IL-13 independent goblet cell hyperplasia in experimental helminth infections. BMC Immunol..

[96] Turner J.-E., Stockinger B., Helmby H. (2013). IL-22 mediates goblet cell hyperplasia and worm expulsion in intestinal helminth infection. PLoS Pathog.

[97] Hasnain S.Z., Evans C.M., Roy M., Gallagher A.L., Kindrachuk K.N., Barron L., Dickey B.F., Wilson M.S., Wynn T.A., Grencis R.K. (2011). Muc5ac: A critical component mediating the rejection of enteric nematodes. J. Exp. Med..

[98] Hasnain S.Z., Dawson P.A., Lourie R., Hutson P., Tong H., Grencis R.K., McGuckin M.A., Thornton D.J. (2017). Immune-driven alterations in mucin sulphation is an important mediator of *Trichuris muris* helminth expulsion. PLoS Pathog..

[99] Artis D., Wang M.L., Keilbaugh S.A., He W., Brenes M., Swain G.P., Knight P.A., Donaldson D.D., Lazar M.A., Miller H.R.P. (2004). RELM/FIZZ2 is a goblet cell-specific immune-effector molecule in the gastrointestinal tract. Proc. Natl. Acad. Sci. USA.

[100] Artis D. (2006). New weapons in the war on worms: Identification of putative mechanisms of immune-mediated expulsion of gastrointestinal nematodes. Int. J. Parasitol..

[101] Nair M.G., Guild K.J., Du Y., Zaph C., Yancopoulos G.D., Valenzuela D.M., Murphy A., Stevens S., Karow M., Artis D. (2008). Goblet cell-derived resistin-like molecule β augments CD4+ T cell production of IFN-γ and infection-induced intestinal inflammation. J. Immunol..

[102] Forman R.A., de Schoolmeester M.L., Hurst R.J.M., Wright S.H., Pemberton A.D., Else K.J. (2012). The goblet cell is the cellular source of the anti-microbial angiogenin 4 in the large intestine post *Trichuris muris* infection. PLoS ONE.

[103] Bell L.V., Else K.J. (2011). Regulation of colonic epithelial cell turnover by IDO contributes to the innate susceptibility of SCID mice to *Trichuris muris* infection. Parasite Immunol..

[104] Coakley G., Harris N.L. (2020). The Intestinal Epithelium at the Forefront of Host–Helminth Interactions. Trends Parasitol..

[105] Worthington J.J., Reimann F., Gribble F.M. (2018). Enteroendocrine cells-sensory sentinels of the intestinal environment and orchestrators of mucosal immunity. Mucosal Immunol..

[106] Cliffe L.J., Humphreys N.E., Lane T.E., Potten C.S., Booth C., Grencis R.K. (2005). Accelerated intestinal epithelial cell turnover: A new mechanism of parasite expulsion. Science.

[107] Motomura Y., Ghia J.-E., Wang H., Akiho H., El-Sharkawy R.T., Collins M., Wan Y., McLaughlin J.T., Khan W.I. (2008). Enterochromaffin cell and 5-hydroxytryptamine responses to the same infectious agent differ in Th1 and Th2 dominant environments. Gut.

[108] Wang H., Steeds J., Motomura Y., Deng Y., Verma-Gandhu M., El-Sharkawy R.T., McLaughlin J.T., Grencis R.K., Khan W.I. (2007). CD4+ T cell-mediated immunological control of enterochromaffin cell hyperplasia and 5-hydroxytryptamine production in enteric infection. Gut.

[109] Manocha M., Shajib M.S., Rahman M.M., Wang H., Rengasamy P., Bogunovic M., Jordana M., Mayer L., Khan W.I. (2013). IL-13-mediated immunological control of enterochromaffin cell hyperplasia and serotonin production in the gut. Mucosal Immunol..

[110] Wang H., Kwon Y.H., Dewan V., Vahedi F., Syed S., Fontes M.E., Ashkar A.A., Surette M.G., Khan W.I. (2019). TLR2 plays a pivotal role in mediating mucosal serotonin production in the gut. J. Immunol..

[111] Kim J.J., Wang H., Terc J.D., Zambrowicz B., Yang Q.M., Khan W.I. (2015). Blocking peripheral serotonin synthesis by telotristat etiprate (LX1032/LX1606) reduces severity of both chemical-and infection-induced intestinal inflammation. Am. J. Physiol. Liver Physiol..

[112] Antignano F., Mullaly S.C., Burrows K., Zaph C. (2011). *Trichuris muris* infection: A model of type 2 immunity and inflammation in the gut. JoVE.

[113] Vallance B.A., Galeazzi F., Collins S.M., Snider D.P. (1999). CD4 T Cells and Major Histocompatibility Complex Class II Expression Influence Worm Expulsion and Increased Intestinal Muscle Contraction during *Trichinella spiralis* Infection. Infect. Immun..

[114] Khan W.I., Vallance B.A., Blennerhassett P.A., Deng Y., Verdu E.F., Matthaei K.I., Collins S.M. (2001). Critical role for signal transducer and activator of transcription factor 6 in mediating intestinal muscle hypercontractility and worm expulsion in *Trichinella spiralis*-infected mice. Infect. Immun..

[115] Vallance B.A., Blennerhassett P.A., Deng Y., Matthaei K.I., Young I.G., Collins S.M. (1999). IL-5 contributes to worm expulsion and muscle hypercontractility in a primary T. spiralis infection. Am. J. Physiol. Liver Physiol..

[116] Khan W.I., Blennerhassett P.A., Deng Y., Gauldie J., Vallance B.A., Collins S.M. (2001). IL-12 gene transfer alters gut physiology and host immunity in nematode-infected mice. Am. J. Physiol. Liver Physiol..

[117] Motomura Y., Khan W.I., El-Sharkawy R.T., Verma-Gandhu M., Grencis R.K., Collins S.M. (2010). Mechanisms underlying gut dysfunction in a murine model of chronic parasitic infection. Am. J. Physiol. Liver Physiol..

[118] Faulkner H., Renauld J.-C., Van Snick J., Grencis R.K. (1998). Interleukin-9 enhances resistance to the intestinal nematode *Trichuris muris*. Infect. Immun..

[119] Chen Z., Luo J., Li J., Kim G., Stewart A., Urban J.F., Huang Y., Chen S., Wu L.-G., Chesler A. (2021). Interleukin-33 Promotes Serotonin Release from Enterochromaffin Cells for Intestinal Homeostasis. Immunity.

[120] Cortes A., Peachey L., Scotti R., Jenkins T.P., Cantacessi C. (2019). Helminth-microbiota cross-talk–A journey through the vertebrate digestive system. Mol. Biochem. Parasitol..

[121] Leung J.M., Graham A.L., Knowles S.C.L. (2018). Parasite-microbiota interactions with the vertebrate gut: Synthesis through an ecological lens. Front. Microbiol..

[122] Hayes K.S., Bancroft A.J., Goldrick M., Portsmouth C., Roberts I.S., Grencis R.K. (2010). Exploitation of the intestinal microflora by the parasitic nematode *Trichuris muris*. Science.

[123] White E.C., Houlden A., Bancroft A.J., Hayes K.S., Goldrick M., Grencis R.K., Roberts I.S. (2018). Manipulation of host and parasite microbiotas: Survival strategies during chronic nematode infection. Sci. Adv..

[124] Leung J.M., Budischak S.A., Chung The H., Hansen C., Bowcutt R., Neill R., Shellman M., Loke P., Graham A.L. (2018). Rapid environmental effects on gut nematode susceptibility in rewilded mice. PLoS Biol..

[125] Holm J.B., Sorobetea D., Kiilerich P., Ramayo-Caldas Y., Estellé J., Ma T., Madsen L., Kristiansen K., Svensson-Frej M. (2015). Chronic *Trichuris muris* infection decreases diversity of the intestinal microbiota and concomitantly increases the abundance of lactobacilli. PLoS ONE.

[126] Houlden A., Hayes K.S., Bancroft A.J., Worthington J.J., Wang P., Grencis R.K., Roberts I.S. (2015). Chronic *Trichuris muris* infection in C57BL/6 mice causes significant changes in host microbiota and metabolome: Effects reversed by pathogen clearance. PLoS ONE.

[127] Ramanan D., Bowcutt R., Lee S.C., San Tang M., Kurtz Z.D., Ding Y., Honda K., Gause W.C., Blaser M.J., Bonneau R.A. (2016). Helminth infection promotes colonization resistance via type 2 immunity. Science.

[128] Duque-Correa M.A., Karp N.A., McCarthy C., Forman S., Goulding D., Sankaranarayanan G., Jenkins T.P., Reid A.J., Cambridge E.L., Reviriego C.B. (2019). Exclusive dependence of IL-10R signalling on intestinal microbiota homeostasis and control of whipworm infection. PLoS Pathog..

[129] D’Elia R., Matthew L.D., Zeef L.A., Wright S.H., Pemberton A.D., Else K.J. (2009). Expulsion of *Trichuris muris* is associated with increased expression of angiogenin 4 in the gut and increased acidity of mucins within the goblet cell. BMC Genomics.

[130] Hamann K.J., Barker R.L., Loegering D.A., Gleich G.J. (1987). Comparative toxicity of purified human eosinophil granule proteins for newborn larvae of *Trichinella spiralis*. J. Parasitol..

[131] Bach J.-F. (2018). The hygiene hypothesis in autoimmunity: The role of pathogens and commensals. Nat. Rev. Immunol..

[132] McKay D.M. (2009). The therapeutic helminth?. Trends Parasitol..

[133] Summers R.W., Elliott D.E., Urban J.F., Thompson R.A., Weinstock J.V. (2005). *Trichuris suis* therapy for active ulcerative colitis: A randomized controlled trial. Gastroenterology.

[134] Reddy A., Fried B. (2007). The use of *Trichuris suis* and other helminth therapies to treat Crohn’s disease. Parasitol. Res..

[135] Vegas-Sanchez M.C., Rollan-Landeras E., Garcia-Rodriguez J.J., Bolas-Fernandez F. (2015). Induction of ulcerative colitis in mice influences the course of infection with the nematode *Trichuris muris*. J. Helminthol..

[136] Elliott D.E., Setiawan T., Metwali A., Blum A., Urban J.F., Weinstock J.V. (2004). Heligmosomoides polygyrus inhibits established colitis in IL 10 deficient mice. Eur. J. Immunol..

[137] Elliott D.E., Li J., Blum A., Metwali A., Qadir K., Urban J.F., Weinstock J.V. (2003). Exposure to schistosome eggs protects mice from TNBS-induced colitis. Am. J. Physiol. Liver Physiol..

[138] Hunter M.M., Wang A., Hirota C.L., McKay D.M. (2005). Neutralizing anti-IL-10 antibody blocks the protective effect of tapeworm infection in a murine model of chemically induced colitis. J. Immunol..

[139] Khan W.I., Blennerhasset P.A., Varghese A.K., Chowdhury S.K., Omsted P., Deng Y., Collins S.M. (2002). Intestinal nematode infection ameliorates experimental colitis in mice. Infect. Immun..

[140] Motomura Y., Wang H., Deng Y., El Sharkawy R.T., Verdu E.F., Khan W.I. (2009). Helminth antigen based strategy to ameliorate inflammation in an experimental model of colitis. Clin. Exp. Immunol..

[141] Sakaguchi S., Yamaguchi T., Nomura T., Ono M. (2008). Regulatory T cells and immune tolerance. Cell.

[142] Bhardwaj E.K., Else K.J., Rogan M.T., Warhurst G. (2014). Increased susceptibility to *Trichuris muris* infection and exacerbation of colitis in Mdr1a-/-mice. World J. Gastroenterol. WJG.

[143] Wilson M.S., Ramalingam T.R., Rivollier A., Shenderov K., Mentink-Kane M.M., Madala S.K., Cheever A.W., Artis D., Kelsall B.L., Wynn T.A. (2011). Colitis and Intestinal Inflammation in IL10−/− Mice Results from IL-13Rα2–Mediated Attenuation of IL-13 Activity. Gastroenterology.

[144] Levison S.E., McLaughlin J.T., Zeef L.A.H., Fisher P., Grencis R.K., Pennock J.L. (2010). Colonic transcriptional profiling in resistance and susceptibility to trichuriasis: Phenotyping a chronic colitis and lessons for iatrogenic helminthosis. Inflamm. Bowel Dis..

[145] Bramhall M., Rich K., Chakraborty A., Logunova L., Han N., Wilson J., McLaughlin J., Brass A., Cruickshank S.M. (2020). Differential expression of soluble receptor for advanced glycation end-products in mice susceptible or resistant to chronic colitis. Inflamm. Bowel Dis..

[146] Chenery A.L., Antignano F., Burrows K., Scheer S., Perona-Wright G., Zaph C. (2016). Low-dose intestinal *Trichuris muris* infection alters the lung immune microenvironment and can suppress allergic airway inflammation. Infect. Immun..

[147] Yazdanbakhsh M., Kremsner P.G., Van Ree R. (2002). Allergy, parasites, and the hygiene hypothesis. Science.

[148] Fleming J.O., Isaak A., Lee J.E., Luzzio C.C., Carrithers M.D., Cook T.D., Field A.S., Boland J., Fabry Z. (2011). Probiotic helminth administration in relapsing-remitting multiple sclerosis: A phase 1 study. Mult. Scler. J..

[149] Osada Y., Shimizu S., Kumagai T., Yamada S., Kanazawa T. (2009). Schistosoma mansoni infection reduces severity of collagen-induced arthritis via down-regulation of pro-inflammatory mediators. Int. J. Parasitol..

[150] Kwon Y.H., Wang H., Denou E., Ghia J.-E., Rossi L., Fontes M.E., Bernier S.P., Shajib M.S., Banskota S., Collins S.M. (2019). Modulation of gut microbiota composition by serotonin signaling influences intestinal immune response and susceptibility to colitis. Cell. Mol. Gastroenterol. Hepatol..

